# A Convenient Synthesis of Amino Acid Methyl Esters

**DOI:** 10.3390/molecules13051111

**Published:** 2008-05-08

**Authors:** Jiabo Li, Yaowu Sha

**Affiliations:** The Key Laboratory of Bioorganic Phosphorous Chemistry and Chemical Biology, Department of Chemistry, Tsinghua University, Beijing 100084, P. R. China; E-mail: lijb05@mails.tsinghua.edu.cn

**Keywords:** Amino acid methyl ester hydrochlorides, amino acids, trimethylchlorosilane, esterification.

## Abstract

A series of amino acid methyl ester hydrochlorides were prepared in good to excellent yields by the room temperature reaction of amino acids with methanol in the presence of trimethylchlorosilane. This method is not only compatible with natural amino acids, but also with other aromatic and aliphatic amino acids.

## Introduction

Amino acid methyl esters are important intermediates in organic synthesis, which have been used in various areas such as peptide synthesis [1], medicinal chemistry [2,3], as chiral sources [4,5,6,7] and polymer materials [8,9].

A variety of reagents have been reported for the transformation of amino acids into amino acid methyl esters, which include protic acids (gaseous hydrochloric acid [10], sulfuric acid and *p*-toluene- sulfonic acid), thionyl chloride [11], 2,2-dimethoxypropane [12] and ion-exchange resins (Amberlyst™-15, [13]). There are other methods which require multistep reactions to obtain the products, such as the sequence of *N*-protection, esterification and deprotection. Although some of them are widely used, they still have several disadvantages, including tedious workup procedures, safety and waste disposal problems and harsh reaction conditions. Methanol/trimethylchlorosilane has been shown to be a convenient system for the preparation of methyl esters of various carboxylic acids [14, 15]. This method has been used in the transformation of *N*-Boc-α-amino acids into *N*-unprotected α-amino methyl esters [16] and some other amino acid methyl esters have been prepared using this system [17,18,19,20]. In order to demonstrate the general applicability of the method, we have examined a series of amino acids as substrates, including natural, aromatic and aliphatic amino acids and in this communication we report that trimethylchlorosilane (TMSCl) with methanol at room temperature is an efficient reagent for esterification of amino acids of all classes. Compared to the methods mentioned above the use of TMSCl/MeOH was more advantageous due to the following features: easy operation, mild reaction conditions, simple workup and good to excellent yields.

## Results and Discussion

**Scheme 1:**
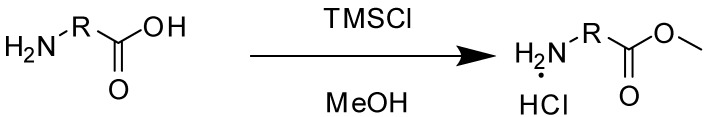


The synthesis of acid methyl ester hydrochlorides is shown in Scheme 1**.** A series of amino acids, including natural amino acids, aromatic amino acids and aliphatic amino acids was transformed to corresponding amino acid methyl ester hydrochlorides in good to excellent yields, which are summarized in Table 1.

Compared with other methods, the yields obtained with the TMSCl/MeOH system were in most cases comparable to or even higher than those obtained with the thionyl chloride/MeOH and HCl(SO_4_H_2_)/MeOH systems and the method is certainly more convenient from an operational point of view. For example, for best results the temperature of the thionyl chloride/MeOH system should be strictly maintained between –5~0 ˚C and HCl gas must be continuously passed through the refluxing mixture in the MeOH/HCl method, making the TMSCl/MeOH system obviously more convenient.

In general, two equivalents of TMSCl were used. However, the substrates in entries 2, 12 and 18 have two carboxyl groups, so four equivalents of TMSCl were used in the esterifications of these substrates. In general the reaction time was 12 h. Because of the poor solubility in methanol of both of substrates and products in entries 1, 2, 13, 14, 19, the reaction time for these substrates was 24 h.

Racemization is a common problem in the synthesis of amino acid esters. According to a published report esterification of protected amino acids by TMSCl showed little racemization [16]. It would of course be more interesting if free amino acids could be directly esterified with little racemization and work to determine if this is possible is currently under way in our lab and the results will be reported in due course.

**Table 1:** Esterification of amino acids witd metdanol in tde presence of TMSCl.

Entry	Substrate	Product ^a^	Time (hr)^b^	Yield (%) ^c^	Reported yields (%) ^d^
1	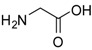	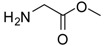	24	96	89 [21]
2	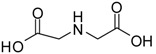	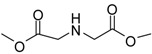	24	89	98 [22]
3	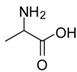	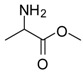	12	97	97 [23]
4	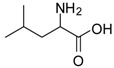	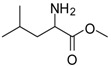	12	96	95 [23]
5	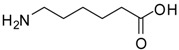	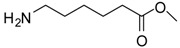	12	88	97 [24]
6	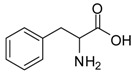	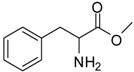	12	96	88 [25]
7	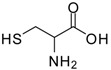	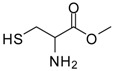	12	85	65 [26]
8	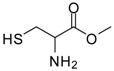	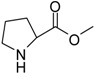	12	76	29 [27]
9	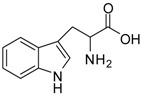	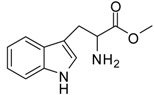	12	91	92 [28]
10	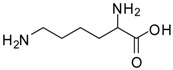	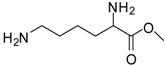	12	94	93 [29]
11	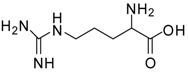	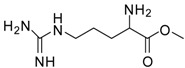	12	89	71 [30]
12	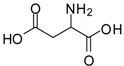	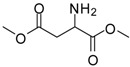	12	86	100 [31]
13	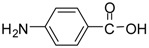	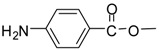	24	86	72 [32]
14	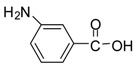	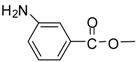	24	90	78 [33]
15	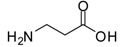	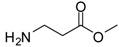	12	96	99 [35]
16	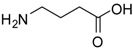	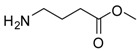	12	86	93 [36]
17	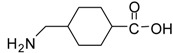	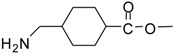	12	94	93 [36]
18	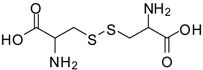	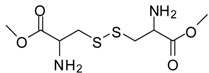	12	98	92 [37]
19	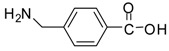	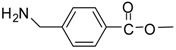	24	97	62 [38]

^a^ All products are isolated as hydrochloride salts.

^b^ Reaction times are not optimized

^c^ All yields refer to isolated products, fully characterized by ^1^H- and ^13^C-NMR and MS.

^d^ The yields of entries 1, 2, 9, 15,16, 17 were with the thionyl chloride/MeOH system, entry 13 in H_2_SO_4_/MeOH system, and the remainder with the HCl/MeOH system.

## Conclusions

We have developed a facile method to synthesize different amino acid methyl ester hydrochlorides through the esterification of corresponding amino acids with methanol using trimethylchlorosilane. The reaction offers convenience, mild conditions and good to excellent yields.

## Experimental

### General

^1^H-NMR (300 MHz) and ^13^C-NMR (75 MHz) spectra were recorded in D_2_O on a JEOL JNM-ECA300 spectrometer. All ESI-MS experiments were done on a Bruker ESQUIRE-LC.

### General procedure for the preparation of amino acid methyl ester hydrochlorides

Amino acid (0.1 mol) was taken in a round bottom flask. Freshly distilled chlorotrimethylsilane (0.2 mol) was added slowly and stirred with a magnetic stirrer. Then methanol (100 mL) was added and the resulting solution or suspension was stirred at room temperature. After the completion of reaction (as monitored by TLC), the reaction mixture was concentrated on a rotary evaporator to give the product amino acid ester hydrochloride.

*Glycine methyl ester (entry 1)*. ^1^H-NMR: δ 4.03 (s, 2H), 3.92 (s, 3H); ^13^C-NMR: δ 168.8, 53.5, 40.2; ESI-MS: calcd. for (M+H)/z: 90.1, found: (M+H)/z: 90.1.

*Dimethyl iminodiacetate (entry 2)*. ^1^H-NMR: δ 4.06 (s, 4H), δ 3.77 (s, 6H); ^13^C-NMR: δ 167.5, 53.6, 47.2; ESI-MS: calcd. for (M+H)/z: 162.1, found: (M+H)/z: 162.3.

*α -Alanine methyl ester* *(entry 3)*. ^1^H-NMR: δ 4.14 (m, 1H), 3.76 (s, 3H), 1.49 (m, 3H); ^13^C-NMR: δ 171.3, 53.7, 48.9, 15.2; ESI-MS: calcd. for (M+H)/z: 104.0, found: (M+H)/z: 104.1.

*Leucine methyl ester (entry 4)*. ^1^H-NMR: δ 4.10 (t, 1H), 3.78 (s, 3H), 1.82 (m, 1H), 1.65 (m, 2H), 0.89 (m, 6H); ^13^C-NMR: δ 171.4, 53.6, 51.6, 38.9, 24.0, 21.6, 21.2; ESI-MS: calcd. for (M+H)/z: 146.0, Found:(M+H)/z: 146.3.

*6-Aminocaproic acid methyl ester (entry 5)*. ^1^H-NMR: δ 3.62 (s, 3H), 2.93 (t, 2H), 2.35 (t, 2H), 1.61-1.55 (m, 4H), 1.33 (m, 2H); ^13^C-NMR: δ 177.4, 52.3, 39.4, 33.5, 26.5, 25.2, 23.8; ESI-MS: calcd. for (M+H)/z: 146.1, found: (M+H)/z: 146.1.

*Phenylalanine methyl ester (entry 6)*. ^1^H-NMR: δ 7.37-7.34 (m, 3H), 7.23 (d, 1H), 7.21 (d, 1H), 4.36 (t, 1H), 3.77 (s, 3H), 3.29-3.13 (m, 2H); ^13^C-NMR: δ 170.1, 133.8, 129.5, 128.2, 54.2, 53.7, 35.7; ESI-MS: calcd. for (M+H)/z: 180.1, found: (M+H)/z: 180.1.

*Methionine methyl ester (entry 7)*. ^1^H-NMR: δ 4.51 (t, 1H), 3.79 (s, 3H), 3.30 (d, 2H); ^13^C-NMR: δ 169.2, 54.0, 51.6, 35.7; ESI-MS: calcd. for (M+H)/z: 139.0, found: (M+H)/z: 139.2.

*Proline methyl ester (entry 8)*. ^1^H-NMR: δ 4.39 (m, 1H), 3.76 (s, 3H), 2.35 (m, 2H), 2.03 (m, 2H), 1.98 (m, 2H); ^13^C-NMR: δ 170.5, 59.7, 53.9, 46.4, 28.4, 23.4; ESI-MS: calcd. for (M+H)/z: 130.0, found: (M+H)/z: 130.1.

*Tryptophan methyl ester (entry 9)*. ^1^H-NMR: δ 7.42 (d, 1H), 7.39 (d, 1H), 7.16 (d, 1H), 7.14 (d, 1H), 7.04 (t, 1H), 4.24 (t, 1H), 3.66 (s, 3H), 3.25 (m, 2H); ^13^C-NMR: δ 170.4, 136.4, 126.5, 125.4, 122.3, 119.6, 118.1, 112.1, 106.0, 53.7, 53.4, 25.7; ESI-MS: calcd. for (M+H)/z: 219.1, found: (M+H)/z: 219.2.

*Lysine methyl ester (entry 10)*. ^1^H-NMR: δ 4.12 (t, 1H), 3.79 (s, 3H), 2.97 (t, 2H), 1.94 (m, 2H), 1.67 (m, 2H), 1.46 (m, 2H); ^13^C-NMR: δ 170.7, 53.7, 52.9, 39.2, 29.4, 26.4, 21.6; ESI-MS: calcd. for (M+H)/z: 161.1, found: (M+H)/z: 161.0.

*Arginine methyl ester (entry 11)*. ^1^H-NMR: δ 4.14 (t, 1H), 3.78 (s, 3H), 3.20 (m, 2H), 1.95 (m, 2H), 1.67 (m, 2H); ^13^C NMR: δ 170.5, 156.9, 53.8, 52.6, 40.4, 27.0, 23.9; ESI-MS: calcd. for (M+H)/z: 189.1, found: (M+H)/z: 189.1.

*Aspartic acid dimethyl ester (entry 12)*. ^1^H-NMR: δ 4.44 (dd, 1H), 3.77 (s, 3H), 3.68 (s, 3H), 3.10 (dd, 2H); ^13^C-NMR: δ 171.7, 169.4, 54.0, 53.1, 49.3, 33.7; ESI-MS: calcd. for (M+H)/z: 162.0, found: (M+H)/z: 162.1.

*Methyl 4-aminobenzoate (entry 13)*. ^1^H-NMR: δ 7.86 (d, 1H), 7.83 (d, 1H), 7.32 (d, 1H), 7.29 (d, 1H), 3.72(s, 3H); ^13^C-NMR: δ 167.9, 135.2, 131.2, 129.6, 123.0, 52.8; ESI-MS: calcd. for (M+H)/z: 152.1, found: (M+H)/z: 152.2.

*Methyl 3-aminobenzoate (entry 14)*. ^1^H-NMR: δ 7.90-7.83 (m, 2H), 7.51 (m, 2H), 3.77 (s, 3H); ^13^C- NMR: δ 167.6, 131.4, 130.6, 130.2, 130.1, 128.0, 123.9, 53.0; ESI-MS: calcd. for (M+H)/z: 152.0, found: (M+H)/z: 152.1.

*β-Alanine methyl ester (entry 15)*. ^1^H-NMR: δ 3.68 (s, 3H), 3.22 (t, 2H), 2.77 (t, 2H); ^13^C-NMR: δ 173.2, 52.7, 35.2, 31.2; ESI-MS: calcd. for (M+H)/z: 104.0, found: (M+H)/z: 104.1.

*γ-Aminobutyric methyl ester (entry 16)*.^ 1^H-NMR: δ 3.63 (s, 3H), 2.97 (t, 3H), 2.45(m, 2H), 1.89 (t, 2H); ^13^C-NMR: δ 175.7, 52.4, 38.8, 30.6, 22.1; ESI-MS: calcd. for (M+H)/z: 118.2, found: (M+H)/z: 118.3.

*Methyl 4-(aminomethyl)cyclohexanecarboxylate (entry 17)*. ^1^H-NMR: δ 3.59 (s, 3H), 2.79 (m, 2H), 2.29 (m, 1H), 1.94-1.90 (m, 2H), 1.78-1.75 (m, 2H), 1.57 (m, 1H), 1.38-1.26 (m, 2H), 1.04-0.92 (m, 2H); ^13^C-NMR: δ 179.4, 52.3, 44.9, 42.6, 34.8, 28.5, 27.7; ESI-MS: calcd. for (M+H)/z: 172.1, found: (M+H)/z: 172.2.

*Cystine dimethyl ester (entry 18)*. ^1^H-NMR: δ 4.50 (t, 1H), 3.79 (s, 3H), 3.37-3.25(m, 2H); ^13^C-NMR: δ 169.2, 54.0, 51.6, 35.7; ESI-MS: calcd. for (M+H)/z: 269.0, found: (M+H)/z: 269.0.

*Methyl 4-(aminomethyl)benzoate (entry 19)*. ^1^H-NMR: δ 7.96 (d, 2H), 7.64 (d, 2H), 4.08(s, 2H), 3.84 (s, 3H); ^13^C-NMR: δ 166.0, 147.8, 130.0, 128.3, 126.6, 51.5, 45.5; ESI-MS: calcd. for (M+H)/z: 166.0, found: (M+H)/z: 166.1.
